# Germline polymorphism of interferon-lambda3 is clinically associated with progression of renal cell carcinoma

**DOI:** 10.18632/oncotarget.23683

**Published:** 2017-12-25

**Authors:** Akinori Nukui, Yoshiaki Yanai, Toyonori Tsuzuki, Hideyuki Abe, Kyoko Arai, Ken-Ichiro Yoshida, Takao Kamai

**Affiliations:** ^1^Department of Urology, Dokkyo Medical University, Mibu, Tochigi, Japan; ^2^Pharmaceutical Marketing Division, Otsuka Pharmaceutical Co. Ltd, Tokushima, Japan; ^3^Department of Surgical Pathology, Aichi Medical University, Nagakute, Japan

**Keywords:** IFN-lambda3, IL-28B, polymorphism, PD-L1, Akt

## Abstract

Renal cell carcinoma (RCC) is an immunogenic tumor that shows a metabolic shift to aerobic glycolysis. The immune system can have opposing host-protective and tumor-promoting effects, and aerobic glycolysis suppresses antitumor immunity. In addition to immunostimulatory effect, increasing numbers of studies have revealed that interferon (IFN) is also involved in promoting immunosuppression. Since various single nucleotide polymorphisms (SNPs) can influence the outcome of anticancer therapy, we investigated SNPs for IFN-lambda3, a new member of IFN family, in 53 patients with metastatic RCC who underwent cytoreductive nephrectomy. The 16 patients who were heterozygous/homozygous for the minor alleles of SNPs for IFN-lambda3 had a significantly worse response to sequential vascular endothelial growth factor-targeting therapy (*P* = 0.0029) and shorter survival (*P* = 0.0033) compared with the 37 patients possessing the major alleles of SNPs for IFN-lambda3. In these 16 patients, the primary tumor showed elevated glucose uptake on positron emission tomography with [18F] fluorodeoxyglucose (*P* = 0.0160) and increased expression of programmed cell death 1 (PD-1)-ligand 1 (PD-L1) and phosphorylated serine/threonine kinase Akt (*P* = 0.0006 and *P* = 0.0043, respectively) compared to the tumors of the patients without these alleles. Since IFN-induced PD-L1 expression on either tumor cells or tumor-infiltrating mononuclear cells can trigger immunosuppression due to crosstalk between cancer cells and T cells, IFN-lambda3 polymorphism might be linked to the immunosuppressive effects of IFNs in cancer. Although this retrospective study lacks mechanistic insight, our findings suggest that IFN-lambda3 polymorphism might be relevant to the progression of RCC.

## INTRODUCTION

Increased angiogenesis and evasion of the host immune system are two mechanisms assisting the proliferation and metastasis of renal cell carcinoma (RCC), and this cancer also displays impairment of oxidative phosphorylation with a metabolic shift to aerobic glycolysis (Warburg effect) [1]. Interactions between tumor cells and the immune system are complex. Although the immune system can suppress tumor development and promote tumor regression, it can also stimulate tumor growth, with these contradictory host-protective and tumor-promoting actions being referred to as cancer immunoediting [2]. Evasion of the host immune response is considered to be one of the “hallmarks of cancer” [3].

It is currently accepted that the immune system can have both host-protective and tumor-promoting actions [4–6]. Interferons (IFNs) are known to have an immunostimulatory effect by enhancing the cross-priming of T cells and driving T cell differentiation [7, 8]. The type III IFNs are members of a new IFN family that consists of IFN-lambda1, IFN-lambda2, and IFN-lambda3, which are also named interleukin (IL)-29, IL-28A, and IL-28B, respectively [9, 10]. The IFN-lambda receptor complex is composed of IL-10 receptor beta (IL-10R beta) and a novel IL-28 receptor alpha (IL-28R alpha) [9, 10]. Signal transduction by type III IFNs seems to be similar to signaling by type I IFNs such as IFN-alpha [7–10]. IFN-alpha is a pleiotropic cytokine belonging to the type I IFNs that binds to the IFN-alpha receptor (IFNAR). There is growing evidence that host antitumor mechanisms are induced by local production of IFNs [7–10]. IFN-alpha promotes the activation of T cells and dendritic cells, as well as antiangiogenic activity including the inhibition of vascular endothelial growth factor (VEGF), which may lead to IFN-induced antitumor immunity. Some patients with metastatic RCC showed a durable antitumor response to IFN-alpha, although the number is small [11]. Similarly, type III IFNs also have antitumor effects [12].

On the other hand, growing studies began revealing that IFNs can also be detrimental by promoting tumor growth; upregulation of nonclassical major histocompatibility complex (MHC) class I molecules that inhibit natural killer (NK) and T cell killing, loss of antigen expression, and induction of immuno-suppressive regulators [13]. We previously reported that increased tumor cell expression of IFNAR and IL-10R is associated with the metastatic potential of RCC and with an unfavorable prognosis [14–16]. In addition, RCC showing higher expression of IFNAR is refractory to IFN-alpha and VEGF-targeting therapy [15, 16]. IFNAR and IL-10R form a gene cluster [17]. Multiple lines evidence support the mechanisms of suppressive effect on antitumor immunity by aerobic glycolysis by crosstalk of cytokines such as IFNs, programmed cell death 1 (PD-1)-ligand 1 (PD-L1), and serine/threonine kinase Akt in the tumor microenvironment, allowing cancers to resist the effects of endogenous tumor-specific T cells [4, 5, 18, 19]. Thus, the IFN signaling can be immunosuppressive and might play a role in progression of cancer.

A patient’s genetic traits can influence the response to anticancer therapy. Sequencing of the human genome and development of high throughput methods for analysis of single nucleotide polymorphisms (SNPs) have made it possible to rapidly investigate a large number of polymorphisms. Certain SNPs located near the IFN-lambda3 gene have been found to influence spontaneous clearance of hepatitis C virus, as well as the response to treatment with pegylated IFN-alpha [20–23]. Against this background, the present study was conducted in Japanese patients with RCC to investigate the association of IFN-lambda3 gene polymorphism with the response to sequential VEGF-targeting therapy and survival, as well as with tumor glucose uptake and expression of PD-L1 and Akt. It was hoped to obtain information on the role of IFN-lambda3 in human RCCs.

## RESULTS

### Characteristics associated with IFN-lambda3 polymorphism

The results of genotyping for three SNPs of IFN-lambda3 (rs8099917, 11881222, and 8103142) in all 53 patients with metastatic RCC (cT_any_N_any_M1) who underwent cytoreductive nephrectomy are summarized in the tables (Tables 1, 2, and Supplementary Table 1). Thirty-seven patients were homozygous for major alleles of the 3 IFN-lambda3 polymorphisms and 16 patients were heterozygous or homozygous for minor alleles of the three SNPs. Among them, only one patient was heterozygous for major alleles of one genotype (rs11881222) and homozygous for major alleles of the other two genotypes (rs8099917 and rs8103142) (Supplementary Table 1). IFN-lambda3 polymorphism was not correlated with the Fuhrman grade, local invasion, regional lymph node involvement, or metastatic site of clear cell RCC (ccRCC) (Table 2 and Supplementary Table 1).

**Table 1:** Polymorphism of IFN-lambda3

Polymorphism		(db SNP ID)	
	rs 8099917	rs 11881222	rs 8103142
Major allele homozygote	T/T	A/A	T/T
Heterozygote	T/G	A/G	T/C
Minor allele homozygote^*^	G/G	G/G	C/C

NCBI (National Center of Biotechnology Information) shows that new attributes have been added to dbSNP to allow searching and filtering of human variation.

Global minor allele frequency (MAF)^*^: dbSNP is reporting the minor allele frequency for each rs included in a default global population. Since this is being provided to distinguish common polymorphism from rare variants, the MAF is actually the second most frequent allele value. In other words, if there are 3 alleles, with frequencies of 0.50, 0.49, and 0.01, the MAF will be reported as 0.49. The current default global population is 1000Genome phase 3 genotype data from 2500 worldwide individuals, released in the May 2013 dataset. (https://www.ncbi.nlm.nih.gov/projects/SNP/docs/rs_attributes.html#gmaf).

Regarding rs8099917: https://www.ncbi.nlm.nih.gov/snp/?term=rs8099917.

Regarding rs 11881222: https://www.ncbi.nlm.nih.gov/snp/?term=rs11881222.

Regarding rs 8103142: https://www.ncbi.nlm.nih.gov/snp/?term=rs8103142.

**Table 2:** Relationship between IFN-lambda3 polymorphism and clinicopathogogical factors

				Response to therapy for metastatic lesions	
				CR/PR/SD>24w (*n* = 24)	SD<24w/PD (*n* = 29)
			Major/Hetero or Minor (*n* = 37)/(*n* = 16)	Major/Hetero or Minor	Major/Hetero or Minor
Grade 1 (*n* = 3)		3	1/2	1/0	0/2
Grade 2 (*n* = 17)		17	14/3	12/0	2/3
Grade 3 (*n* = 23)		23	14/9	7/3	7/6
Grade 4 (*n* = 9)		10	8/2	1/0	7/2
		53	37/16	21/3	16/13
pT1N0M1 (*n* = 5)	Pul	2	2/0	2/0	
	Pul + Oss	3	2/1	2/1	
		5	4/1	4/1	0/0
pT2N0M1 (*n* = 8)	Pul	3	2/0	2/0	0/1
	Hep	1	1/0		1/0
	Oss	1	1/0		1/0
	Pul + Oss	2	2/0	1/0	1/0
	Pul + Lym	1	0/1		0/1
		8	6/2	3/0	3/2
pT3N0M1 (*n* = 36)	Pul	18	13/5	9/2	4/3
	Lym	1	1/0	1/0	
	Pul + Oss	8	4/4	2/0	2/4
	Pul + Hep	7	5/2	1/0	4/2
	Pul + Lym	1	0/1		0/1
	Pul + Lym + Oss	1	1/0		1/0
		36	24/12	13/2	11/10
pT4NanyM1 (*n* = 4)	Pul + Oss	1	1/0		1/0
	Lym + Oss	1	1/0	1/0	
	Pul + Hep + Oss + Lym	1	0/1		0/1
	Pul + Hep + Oss	1	1/0		1/0
		4	3/1	1/0	2/1
		53	37/16	21/3	16/13

Major : Major homozygote, Hetero : Heterozygote, Minor : Minor homozygote.

CR/PR/SD>24 m : complete, partial, or stable with >24 weeks response.

SD<24m/PD : stable disease for <24 weeks or progressive disease.

Metastatic lesions*; Hep; Liver, Lym; lymph node, Oss; Bone, Pul; Lung.

### Association of IFN-lambda3 polymorphism with the response to VEGF-targeting therapy and the prognosis

After cytoreductive nephrectomy, the 53 patients received sunitinib or pazopanib as first-line therapy for extra-renal metastases. Fifteen patients showed a favorable response [complete response (CR), partial response (PR), or stable disease for >24 weeks (SD>24w)] to sunitinib or pazopanib, while 38 patients had an unfavorable response [stable disease for <24 weeks (SD<24w) or progressive disease (PD)] and subsequently received axitinib as second-line therapy. Among these 38 patients, 9 patients showed a favorable response to axitinib.

When the 24 patients with a favorable response to sunitinib or pazopanib (*n* = 15) or axitinib (*n* = 9) were combined in a favorable response group, those who were heterozygous/homozygous for minor alleles of the SNPs showed a worse response to sequential VEGF-targeting therapy than those who were homozygous for the major alleles (*P* = 0.0029, Table 3).

**Table 3:** Relationship between IFN-lambda3 polymorphism and treatment response for metastatic renal cell carcinoma (*n* = 53)

	CR/PR/SD>24w (*n* = 24)	SD<24w/PD (*n* = 29)	*p* value
Heterozygote or Minor homozygote (*n* =16)	3	13	0.0029
Major homozygote (*n* =37)	21	16	

**Figure 1:**
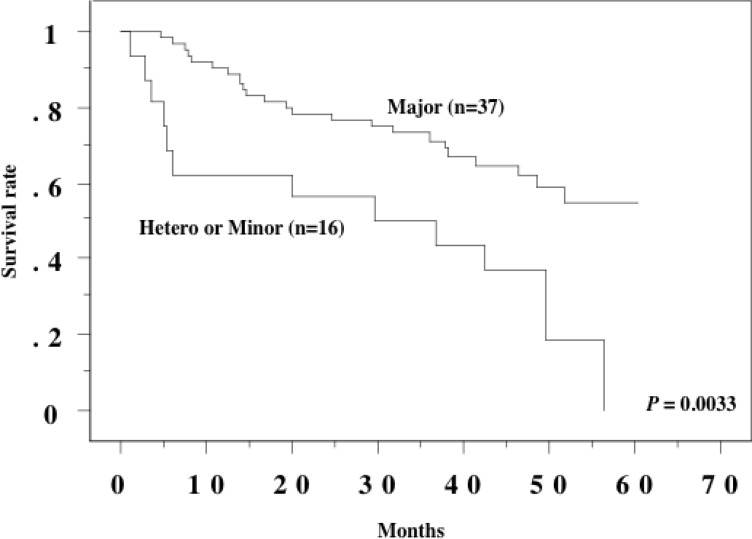
Overall survival curve in 53 metastatic RCCs Patients with heterozygote or minor homozygote for IFN-lambda3 polymorphisms showed shorter overall survival than those with major homozygote.

Kaplan-Meier analysis revealed that patients who were heterozygous/homozygous for minor alleles of the SNPs had a shorter overall survival time than those who were homozygous for the major alleles (*P* = 0.0033, Figure 1).

### Association of IFN-lambda3 polymorphism with glucose uptake and expression of Akt and PD-L1

**Figure 2:**
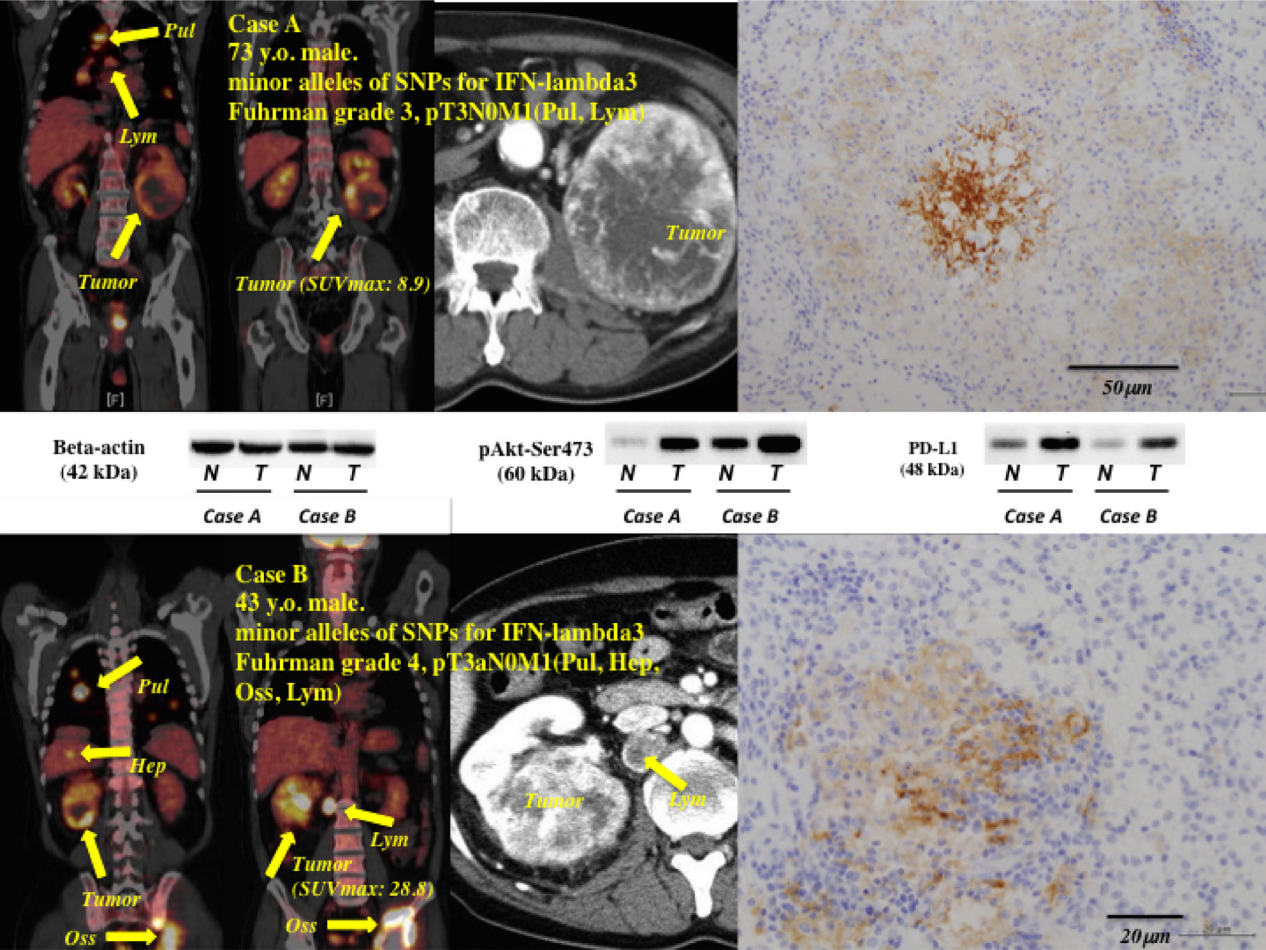
Representative images of PET-CT accompanied with the data of IFN-lambda3 polymorphism, pAkt, and PD-L1 Case (**A**) and case (**B**) showed minor alleles of SNPs for IFN-lambda3 polymorphism. Both patients showed poorer response for first-line sunitinib and second-line axitinib. SUVmax value for the primary tumor was 8.9 in case A, and 28.8 in case B. Expression levels of phosphorylated Akt(Ser-473) and PD-L1 in the primary tumors were higher than those in non-tumor tissues by western blotting. Immunohistochemical study for PD-L1 showed moderate to intense staining. Pul; Lung, Hep; Liver, Oss; Bone, Lym; lymph node in mediastinum (case A) or retroperitoneum (case B).

The representative images of positron emission tomography (PET) with [18F] fluorodeoxyglucose (^18^F-FDG-PET)/computed tomography (CT) accompanied with data of with the data of SNP of IFN lambdha3, phosphorylated Akt (Ser-473) (pAkt(Ser-473)), and PD-L1 were shown in Figure 2.

**Figure 3:**
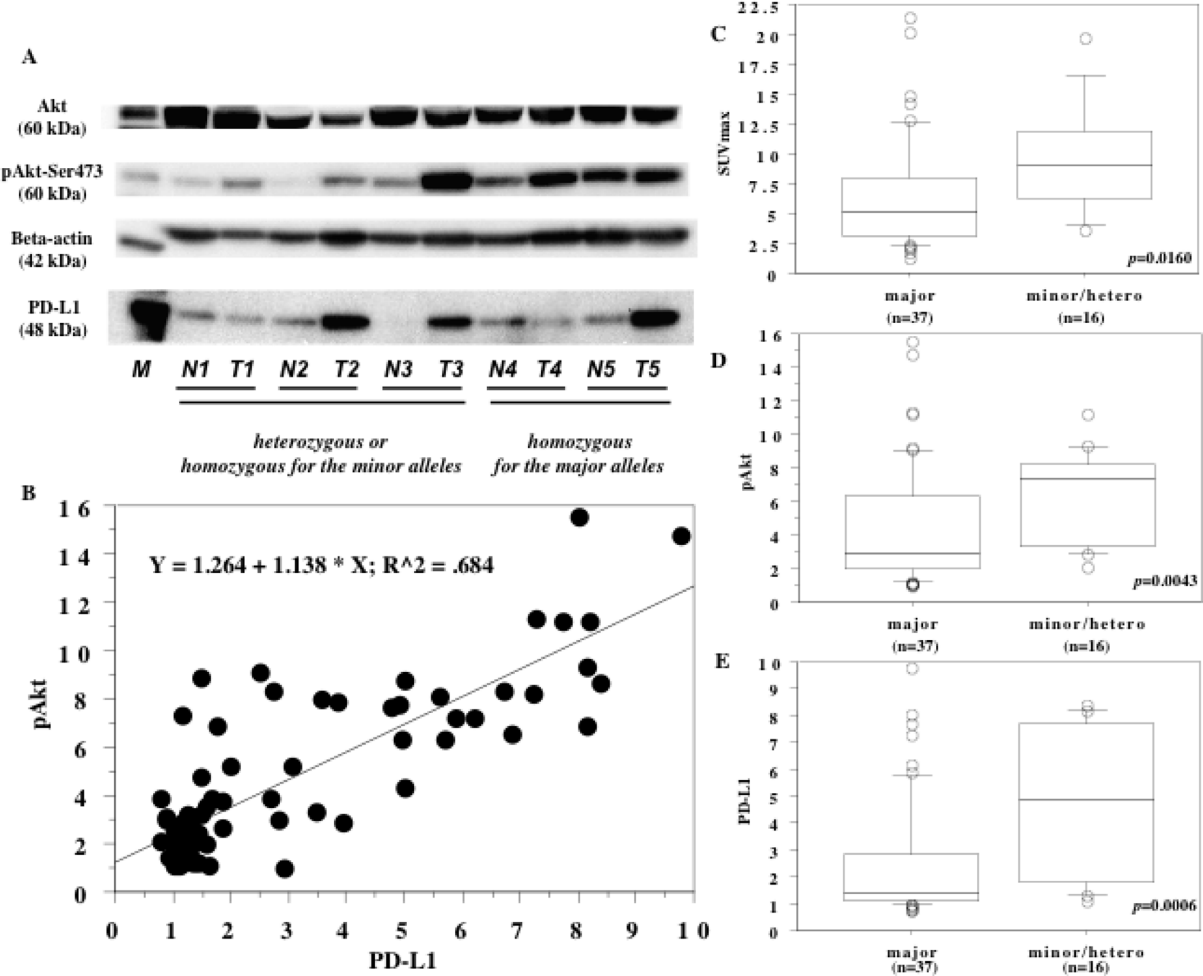
Results of western blotting and influence of IFN-lambda3 polymorphism (**A**) Expression of phosphorylated Akt(Ser-473) (60 kDa), Akt (60 kDa), PD-L1 (48 kDa), and beta actin (42 kDa) proteins in primary tumor tissues. M; marker. N; non-tumor tissue. T; primary tumor. Each number designates a patient. (**B**) Spearman’s rank correlation coefficient analysis. There was a positive correlation between expression of PD-L1 and expression of phosphorylated Akt(Ser-473) in the primary tumors. Patients who were heterozygous/homozygous for the minor alleles of the SNPs had a higher preoperative SUVmax (**C**) and higher expression of pAkt(Ser-473) (**D**) and PD-L1 (**E**). The central line indicates the median value, the box shows the interquartile range, the bars display the full range, and the points are outliers (C–E). Ratio of the optical density of the tumor specimen to that of the corresponding non-neoplastic specimen (set at 1.0) in western blotting for pAkt (D) and PD-L1 (E).

Expression of pAkt(Ser-473) and PD-L1 was detected in both tumor tissues and non-tumor tissues by western blotting (Figure 3A). It was found that pAkt(Ser-473) and PD-L1 showed significantly higher expression in tumor tissues (mean ± S.D. = 2.5 ± 3.4 and 3.0 ± 2.5, respectively) compared with non-tumor tissues (set at 1.0), (*P* < 0.0001, *P* < 0.0001, respectively). In contrast, there was no difference of Akt expression between non-tumor and tumor tissues (*P* = 0.6347, Figure 3A).

There was a positive correlation between expression of PD-L1 and expression of pAkt(Ser-473) in the primary tumor (*r*^2^ = 0.68, *P* = 0.0001, Figure 3B). In the patients who were heterozygous/homozygous for minor alleles of the SNPs, the preoperative maximum standardized uptake value (SUVmax), pAkt(Ser-473) expression, and PD-L1 expression were all significantly increased compared to patients without these alleles (*P* = 0.0160, *P* = 0.0043, and *P* = 0.0006, respectively, Figure 3C–3E).

**Figure 4:**
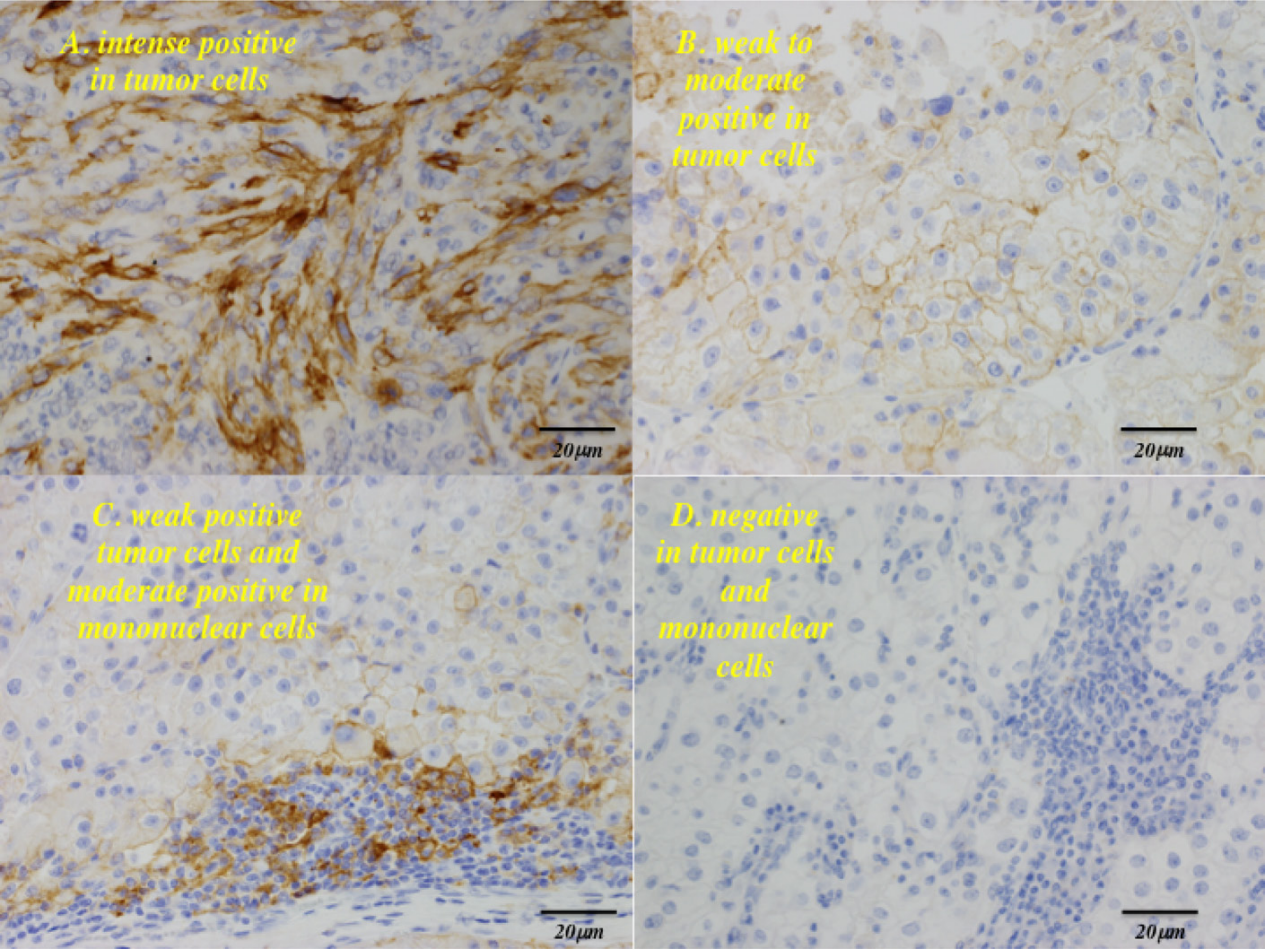
PD-L1 expression of formalin-fixed paraffin-embedded samples immunostained with anti–PD-L1 antibody Representative images of four primary ccRCC tumors (×400). Membranous expression of PD-L1 in tumor cells is detected in the primary tumor (**A** and **B**). Positive membranous staining for PD-L1 is present in both tumor cells and mononuclear cells (**C**). Negative staining for PD-L1 in tumor cells and mononuclear cells (**D**).

When immunohistochemistry was performed, 41 out of 53 specimens (77%) had some degree tumor cell membrane staining for PD-L1 (Figure 4). Corresponding to the results of western blotting, elevated immunostaining for PD-L1 was detected in the 16 patients who were heterozygous/homozygous for minor alleles of the three SNPs (*P* = 0.0014, Supplementary Table 2). The intensity tumor cell membrane immunostaining for PD-L1 was positively correlated with SUVmax (*P* < 0.0001), and with the levels of pAkt(Ser-473) and PD-L1 expression shown by western blotting (*P* < 0.0001 and *P* < 0.0001, respectively, Supplementary Figure 1).

### Relation of IFN-lambda3 polymorphism to survival in all RCC patients

According to Cox univariate analysis, IFN-lambda3 polymorphism, PD-L1 expression, pAkt(Ser-473) expression, SUVmax, and histological grade were all associated with shorter overall survival, but only PD-L1 and histological grade were significant determinants according to multivariate analysis (Table 4).

**Table 4:** Cox regression analysis for various potential prognostic factors in overall survival

Variable	Unfavorable/ favorable characteristics	No. of Patients	Univariate (U)			Multivariate (M)		
			Relative risk	95% confidential interval	*P* value	Relative risk	95% confidential interval	*P* value
IFN-lambda3	heter or minor/major	16/37	2.618	1.278–5.366	0.0086	2.811	0.752–10.511	0.1246
PD-L1	high/low	26/27	4.918	2.078–8.480	0.00003	3.891	1.205–6.479	0.0266
pAkt	high/low	26/27	2.601	1.116–6.058	0.0267	1.715	0.348–8.456	0.5075
SUVmax	high/low	26/27	3.105	1.711–5.634	0.00008	3.013	0.285–31.844	0.3593
Grade	4/3/2/1	5/19/25/4	3.963	2.009–7.815	0.0002	4.166	1.051–3.980	0.0409
pT	4, 3/2, 1	31/22	2.501	0.863–7.248	0.0913			

## DISCUSSION

The present retrospective study identified several differences between patients who were heterozygous/homozygous for the minor alleles of IFN-lambda3 SNPs and patients who were homozygous for the major alleles, with the heterozygous/homozygous the minor alleles patients showing 1) an unfavorable response to sequential VEGF-targeting therapy, 2) a worse prognosis, 3) elevation of SUVmax in the primary tumor, and 4) increased expression of pAkt(Ser-473) and PD-L1 in the primary tumor. These findings suggested that heterozygosity or homozygosity for the minor alleles of IFN-lambda3 SNPs may be clinically associated with increased tumor aerobic glycolysis, immunosuppression, tumor resistance to therapy, and poor survival of patients with RCC. IFNs produced by T cells in the tumor microenvironment induce PD-L1 expression by tumor cells, allowing the tumor to evade immune attack by inhibiting T cell activity through interaction with PD-1 [4, 5]. Therefore, our findings suggest that particular germline polymorphisms of IFN-lambda3 might be linked with the immunosuppressive effect of IFNs in cancer patients.

It has been reported that cancer cells can evade immune surveillance by expressing PD-L1 and by negative regulation of the host immune response [24]. A strong association between higher tumor PD-L1 expression and adverse clinical outcomes was reported in RCC patients receiving anti-VEGF targeting therapy [6], leading to a focus on PD-1/PD-L1 blockade as a possible strategy for antitumor immunotherapy in patients with advanced RCC [25]. In the present study, PD-L1 expression by the primary tumor was higher in patients who were heterozygous/homozygous for the minor alleles of IFN-lambda3 polymorphisms, and their tumors showed resistance to sequential VEGF-targeting therapy. Because elevated tumor expression of PL-L1 was an independent prognostic factor for shorter overall survival in patients with ccRCC, the level of PD-L1 expression by the primary tumor may be partly influenced by IFN-lambda3 polymorphism.

Tumor tissues have miscellaneous components such as tumor cells, immune cells, extracellular matrix, and fibroblasts. Immunohistochemical staining can reveal the site/location of expression or the protein-expressing cells. In the present study, we showed that PD-L1 was expressed on the cell membranes of some tumor cells and tumor-infiltrating mononuclear cells by immunohistochemical staining, and the intensity of PD-L1 immunostaining on tumor cell membranes was positively related to the level of PD-L1 expression shown by western blotting. In fact, PD-L1 protein expression on the cell surface was recently demonstrated by western blotting [26]. Therefore, the band detected by western blotting in the present study might represent total PD-L1 expression by both tumor cells and tumor-infiltrating mononuclear cells. In addition, the tumor-infiltrating mononuclear cells expressing PD-L1 may be tumor antigen-specific cells responding to the cancer. Thus, upregulation of PD-L1 in the tumor microenvironment seems to play a pivotal role in the resistance of cancer to elimination by endogenous tumor-specific T cells [4–6].

Two different mechanisms regulating PD-L1 expression in the tumor microenvironment have been suggested in relation to innate and adaptive immune resistance [4, 5]. The mechanism of innate immune resistance involves a link between PD-L1 and Akt, since constitutive activation of oncogenic signaling systems, such as the Akt pathway, up-regulates PD-L1 expression on tumor cells independently of inflammatory signals in the tumor microenvironment [4, 5]. In the present study, we identified a positive relationship between PD-L1 and pAkt(Ser-473) expression in the primary tumors. Cancer cells are thought to rely on aerobic glycolysis to meet elevated energy requirements for growth and proliferation. A recent study showed that constitutive activation of the Akt/mTOR pathway in tumor cells leads to increased glycolysis and elevated uptake of extracellular glucose, with resultant depletion of extracellular glucose causing dysfunction of tumor-infiltrating T cells and suppression of antitumor immunity [18, 19]. Chang *et al.* reported that PD-L1 expression by tumor cells promotes glycolysis through constitutive activation of the Akt/mTOR pathway [18]. Importantly, Akt activation may promotes glucose utilization to provide energy for cell proliferation and facilitate energy metabolism and cell survival [27, 28], and could potentially increase VEGF production because Akt signaling induces tumor angiogenesis by regulating VEGF via both hypoxia-inducible factor (HIF)-dependent and -independent mechanisms [29, 30]. The present study demonstrated that increased PD-L1 expression was associated with elevation of SUVmax and higher expression of pAkt(Ser-473). Moreover, an increase of HIF-1 was reported to be related to elevation of PD-L1 expression and down-regulation of T cell function [31, 32]. Since RCC is HIF/VEGF-dependent tumor [11], it is likely that increased expression of HIF-1 can result in immunosuppression in addition to promoting the proliferation of RCC.

The detrimental role of IFNs in cancer progression has not been fully elucidated, but several studies have provided evidence of adaptive resistance through IFN-driven induction of PD-L1, which can trigger immunosuppression by crosstalk between cancer cells and tumor-infiltrating mononuclear cells (macrophages, myeloid-derived suppressor cells, dendritic cells, or even lymphocytes) in the tumor microenvironment [4, 5]. PD-L1 suppresses T cell migration/proliferation and also restricts cancer cell killing by binding to T cell receptors (TCRs) [4, 5]. In the tumor microenvironment, expression of co-inhibitory receptors such as PD-1 downregulates T cell activation via specific TCRs and inhibits T cell effector functions [6]. In addition to depending on TCR activation, the extent and duration of PD-1 expression by T cells is regulated via IFNAR signaling, which means that IFNAR signaling weakens T cell responses in the context of continuous T cell activation (e.g., during chronic infection or cancer) [33]. In the present study, RCC patients who were heterozygous or homozygous for the minor alleles of IFN-lambda3 polymorphisms showed increased expression of pAkt(Ser-473) and PD-L1. The process of signal transduction by type III IFNs may be similar to that by type I IFN-alpha [7–10]. Based on the concepts of innate and adaptive resistance [4, 5], PD-L1 might be a convergence point for the IFN pathways and the Akt/mTOR pathway, and these pathways may create a vicious cycle in the tumor microenvironment of RCC patients. In order to understand the role of IFN-lambda3 in more detail, it would be necessary to examine the relationship of IFN-lambda3 polymorphisms with IFN-lambda3 mRNA and protein levels, and the direct association of between IFN-lambda3 polymorphism with increased PD-L1 or pAkt(Ser-473) expression in ccRCC patients.

The limitations of the present study were its retrospective design, a relatively small patient population, and a follow-up period that was too short to allow definite conclusions to be drawn. The main limitation of this study is the lack of any mechanistic insight with respect to how IFN-lambda3 polymorphisms regulate PD-L1 expression. Since PD-L1 suppresses the activity of T cells expressing PD-1, the level of PD-1 expression may also be important in this context. Moreover, it may be worthwhile to determine the molecular mechanisms through which PD-L1 expressed by tumor cells and/or tumor-infiltrating mononuclear cells acts cooperatively or independently to trigger immunosuppression in the tumor microenvironment. Finally, we only investigated three SNPs of IFN-lambda3 (rs8099917, 11881222, and 8103142) because we did not have the ability to analyze other SNPs, but examining other SNPs of IFN-lambda3 (as well as IFN-lambda1, IFN-lambda2, IL-10R beta, and IL-28R alpha) in the future could increase understanding of the role of IFN-lambdas and their receptors in RCC. In order to determine what IFN-lambda3 polymorphisms actually mean and how these polymorphisms interact with other factors that influence immunity and/or energy metabolism in RCC patients, we need to study the mechanisms underlying the relationship between IFN-lambda3 polymorphism and tumor sensitivity to VEGF-targeting or PD-1/PD-L1 blockade therapies and/or overall survival, including immune cell infiltration and angiogenesis, the systemic immune response and cytokine profile, and variation of the tumor response to cytokines associated with different SNPs. Without such information we cannot determine whether these polymorphisms are actually relevant to PD-L1-mediated immunosuppression. The results of such studies might be able to shed more light on the role of IFN-lambda3 signaling in human cancer.

In conclusion, the present retrospective study of RCC patients showed that particular germline polymorphisms of IFN-lambda3 were associated with an unfavorable response to sequencing VEGF-targeting therapy and worse overall survival. These findings suggest that IFN-lambda3 polymorphism might be biologically relevant to the progression of RCC.

## MATERIALS AND METHODS

### Patients

This was a retrospective investigation of IFN-lambda3 polymorphism in 53 patients (33 men and 20 women) who had histopathologically confirmed ccRCC with metastasis (cT_any_N_any_M1) and underwent cytoreductive nephrectomy at our center between 2011 and 2014 before receiving any other therapy. None of the patients had inflammatory and/or autoimmune diseases. Tumor grade and clinical stage were determined according to the Fuhrman system and the TNM classification, respectively [34, 35]. The postoperative follow-up period ranged from 3 to 63 months (median: 31 months).

Inclusion criteria were as follows: 1) age ≥18 years; 2) histologically confirmed diagnosis of ccRCC; 3) cytoreductive nephrectomy and subsequent first-line therapy for metastasis with sunitinib (initial dose of 37.5 or 50 mg/day and four weeks on/two weeks off therapy) or pazopanib (initial dose of 600 or 800 mg/day) that was discontinued due to disease progression, non-response without progression, or intolerance; 4) subsequent second-line therapy with axitinib (recommended starting dose of 10 mg/day); and 5) complete medical records for the entire period from initiation of first-line therapy until the most recent follow-up or death. Progression was determined by the attending physician based on radiographic evidence of tumor enlargement or occurrence of new lesions, worsening of performance status, or exacerbation of cancer-related symptoms (e.g., pain, fever, and weight loss). The doses of sunitinib, pazopanib, and axitinib were decreased if grade 3/4 toxicity occurred. The response to treatment was assessed according to RECIST criteria, with final assessment being performed by review of the medical records in March 2016.

### Genotyping for IFN-lambda3 polymorphism

This study was conducted in accordance with the Helsinki Declaration and was approved by the ethical review board of Dokkyo Medical University Hospital. Each patient signed a consent form before undergoing cytoreductive nephrectomy that was approved by our institutional Committee on Human Rights in Research. Extraction of genomic DNA from whole blood samples and genotyping for three SNPs of IFN-lambda3 (rs8099917, 11881222, and 8103142) with the InvaderPlus assay was carried out by SRL Inc. (Tokyo, Japan), as described previously [36]. The polymorphisms detected near the IFN-lambda3 gene are summarized in Table 1.

### ^18^F-FDG-PET

In all patients, ^18^F-FDG-PET/CT was done for preoperative staging prior to nephrectomy, and the SUVmax was calculated and was defined as the baseline SUVmax.

### Western blotting

Both tumor tissue (from three different sites) and various non-neoplastic kidney tissues were obtained from each patient. The tumor and non-neoplastic specimens were carefully dissected free of stromal tissue. Since inter-individual variations in the expression of pAkt(Ser-473) and PD-L1 may be important, tumor tissue samples and the corresponding non-neoplastic tissues from the same patient were compared to compensate for variation of pAkt(Ser-473) expression. The following antibodies were employed: an anti-rabbit monoclonal antibody for phosphorylated Akt (Ser-473) and an anti-rabbit monoclonal antibody for Akt (Cell Signaling Technology, Inc.; PhosphoPlus Akt (Ser-473) Antibody Kit; # 9270, Danvers, MA), a rabbit anti-human antibody targeting PD-L1 (Cell Signaling Technology, Inc.; PD-L1 (E1L3N) XP Rabbit mAb; # 13684, Danvers, MA), and an antibody for beta-actin (Millipore; # 1501R, Bedford, MA).

Bands of antibody-bound proteins were visualized by chemiluminescence. Then the membrane was scanned for densitometry with a PDI imaging scanner (Agfa Japan, Tokyo) and the data were analyzed with ImageJ for Mac OS (version 1.50), a public domain, Java-based image processing program developed at the National Institutes of Health. Expression of phosphorylated Akt (Ser-473), Akt, and PD-L1 was calculated relative to that of beta-actin in the tumor tissue specimens and corresponding normal tissue specimens. For quantification of these proteins, the relative amount of phosphorylated Akt (Ser-473), Akt, and PD-L1 in tumor tissue specimens was expressed as a ratio of the optical density for the tumor specimen to that for the corresponding non-neoplastic specimen (set at 1.0) by densitometric analysis, as described previously [16]. Mean values for the three tumor tissue samples were used for analysis as described previously [16]. Scoring of the level of expression was done independently by two authors (AN and TK).

### Immunohistochemistry

To support the data obtained by western blot analysis, immunohistochemistry for PD-L1 was performed with the same antibody utilized for western blotting. Formalin-fixed, paraffin-embedded tumor samples were retrieved for examination. The primary anti-PD-L1 antibody was used with an OMNIS automated stainer (Agilent Technology, Santa Clara, CA) and proprietary reagents according to the manufacturer’s protocol. PD-L1 expression on tumor cell membranes was determined semiquantitatively as described previously [25]: 0 (no appreciable staining), 1+ (weak staining), 2+ (moderate staining), and 3+ (strong staining). In each patient, PD-L1 staining was scored by assessing 500 to 1000 cancer cells in 5–10 microscopic fields of 3 to 7 sections at ×400 magnification, as reported previously [37]. The sections were scored independently by three authors (AN, TT, and TK).

### Statistical analysis

Associations between the response to VEGF-targeting therapy and IFN-lambda3 polymorphism were analyzed by Pearson’s χ^2^ test for contingency tables. The Mann-Whitney *U* test was used to compare two groups. Spearman’s rank correlation coefficient analysis was employed to determine the relation between expression of PD-L1 and expression of pAkt(Ser-473). The median SUVmax value, pAkt(Ser-473) expression, and PD-L1 expression in tumor tissues was 5.9, 3.2, and 1.5, respectively. The patients were divided into two groups (a high-expression group and a low-expression group) at these cut-off values.

Cause-specific survival curves were drawn by the Kaplan-Meier method and differences of survival were examined by the log-rank test. The impact of IFN-lambda3 polymorphism, PD-L1, pAkt(Ser-473), SUVmax, histological grade, pT stage, and metastasis on survival was assessed by Cox proportional hazards analysis using univariate and multivariate models. In all analyses, *P* < 0.05 was considered to indicate significance. Data were analyzed with commercially available software [16].

## SUPPLEMENTARY MATERIALS FIGURE AND TABLES


