# Supplementary material for: Germline polymorphism of interferon-lambda3 is clinically associated with progression of renal cell carcinoma
Source: Oncotarget. 2017 Dec 25;9(3):4188–99. doi: 10.18632/oncotarget.23683 (PMC5790531; doi:10.18632/oncotarget.23683)
Supplement: Supplementary file 1 [file oncotarget-09-4188-s001.pdf]

Germline polymorphism of interferon-lambda3 is clinically associated with progression of renal cell carcinoma

SUPPLEMENTARY MATERIALS

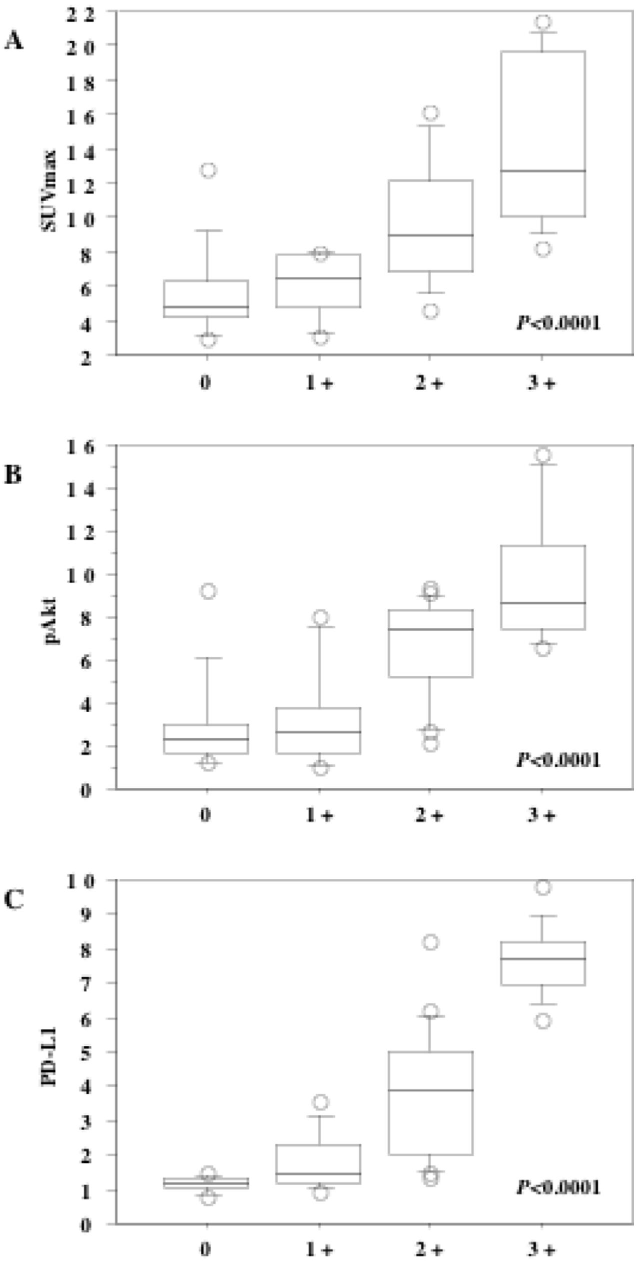

Supplementary Figure 1: Relationship between immunostaining intensity for PD-L1 and SUVmax, and expression levels of phosphorylated Akt(Ser-473) and PD-L1 shown by western blotting. The tumors with intense immunostainig of PD-L1 showed higher preoperative SUVmax (**A**), and increased expression of pAkt(Ser-473) (**B**) and PD-L1 (**C**). X–axis is intensity of immunestaining of PD-L1. Y-axis for pAkt and PD-L1 is a ratio of the optical density for the tumor specimen to that for the corresponding non-neoplastic specimen (set at 1.0) by western blotting.

Supplementary Table 1A: Allele detection of polymorphisms near the IFN-lambda3 gene in all patients

Fuhrman Grade	pTNM stage	single nucleotide polymorphisms (SNPs) ID			Response to agents	Outcome
		rs8099917	rs11881222	rs8103142		
3	pT3aN0M1(Pul)	Major	Major	Major	CR/PR/SD>24w	AWD
3	pT2aN0M1(Hep)	Major	Major	Major	SD<24w/PD	DOD
3	pT4N0M1(Pul, Lym)	Major	Major	Major	CR/PR/SD>24w	AWD
3	pT1aN0M1(Pul, Oss)	**Hetero**	**Hetero**	**Hetero**	CR/PR/SD>24w	DOD
2	pT3aN0M1(Pul)	Major	Major	Major	SD<24w/PD	DOD
2	pT3aN0M1(Pul)	Major	Major	Major	CR/PR/SD>24w	DOD
3	pT3aN0M1(Pul, Lym)	**Minor**	**Minor**	**Minor**	SD<24w/PD	AWD
2	pT3aN0M1(Pul)	Major	Major	Major	CR/PR/SD>24w	AWD
2	pT1aN0M1(Pul, Oss)	Major	Major	Major	CR/PR/SD>24w	AWD
3	pT3bN0M1(Pul, Hep)	Major	Major	Major	CR/PR/SD>24w	DOD
3	pT3aN0M1(Pul)	**Hetero**	**Hetero**	**Hetero**	CR/PR/SD>24w	AWD
2	pT2aN0M1(Pul, Oss)	Major	Major	Major	SD<24w/PD	DOD
1	pT1aN0M1(Pul)	Major	Major	Major	CR/PR/SD>24w	AWD
3	pT3aN0M1(Pul)	Major	**Hetero**	Major	CR/PR/SD>24w	DOD
3	pT3aN0M1(Pul)	**Hetero**	**Hetero**	**Hetero**	SD<24w/PD	DOD
2	pT3aN0M1(Pul)	Major	Major	Major	CR/PR/SD>24w	AWD
2	pT2aN0M1(Pul)	Major	Major	Major	CR/PR/SD>24w	AWD
3	pT3bN0M1(Pul, Hep)	**Hetero**	**Hetero**	**Hetero**	SD<24w/PD	DOD
3	pT3bN0M1(Pul)	Major	Major	Major	SD<24w/PD	DOD
4	pT3bN0M1(Pul, Oss, Lym)	Major	Major	Major	SD<24w/PD	DOD
4	pT3aN0M1(Pul, Hep)	Major	Major	Major	SD<24w/PD	DOD
2	pT2aN0M1(Pul, OSS)	Major	Major	Major	CR/PR/SD>24w	AWD
2	pT3bN0M1(Pul)	**Hetero**	**Hetero**	**Hetero**	SD<24w/PD	DOD
3	pT3aN0M1(Pul, Hep)	Major	Major	Major	SD<24w/PD	DOD
3	pT3aN0M1(Pul, Hep)	Major	Major	Major	SD<24w/PD	DOD
3	pT3aN0M1(Pul, Oss)	Major	Major	Major	CR/PR/SD>24w	DOD
3	pT3aN0M1(Pul, Oss)	**Hetero**	**Hetero**	**Hetero**	SD<24w/PD	DOD
2	pT3aN0M1(Pul)	Major	Major	Major	CR/PR/SD>24w	AWD
2	pT3aN0M1(Pul, Oss)	**Hetero**	**Hetero**	**Hetero**	SD<24w/PD	DOD
3	pT3aN0M1(Pul)	Major	Major	Major	CR/PR/SD>24w	DOD
3	pT3aN0M1(Pul. Hep)	Major	Major	Major	SD<24w/PD	DOD
3	pT3aN0M1(Pul, Oss)	Major	Major	Major	SD<24w/PD	DOD
4	pT2aN0M1(OSS)	Major	Major	Major	SD<24w/PD	DOD
3	pT2aN0M1(Pul)	**Hetero**	**Hetero**	**Hetero**	SD<24w/PD	DOD
3	pT2aN0M1(Pul)	Major	Major	Major	CR/PR/SD>24w	AWD
2	pT3bN0M1(Pul)	Major	Major	Major	CR/PR/SD>24w	DOD
2	pT1bN0M1(Pul)	Major	Major	Major	CR/PR/SD>24w	AWD
2	pT3aN0M1(Pul)	**Minor**	**Minor**	**Minor**	SD<24w/PD	AWD

Supplementary Table 1B: Allele detection of polymorphisms near the IFN-lambda3 gene in all patients

Fuhrman Grade	pTNM stage	single nucleotide polymorphisms (SNPs) ID			Response to agents	Outcome
		rs8099917	rs11881222	rs8103142		
1	pT3aN0M1(Pul, Oss)	**Hetero**	**Hetero**	**Hetero**	SD<24w/PD	DOD
3	pT3bN0M1(Lym)	Major	Major	Major	CR/PR/SD>24w	DOD
4	pT4N1M1(Lym, Oss)	Major	Major	Major	SD<24w/PD	DOD
3	pT3bN0M1(Pul)	Major	Major	Major	SD<24w/PD	DOD
2	pT3aN0M1(Pul, Oss)	Major	Major	Major	CR/PR/SD>24w	DOD
1	pT3aN0M1(Pul, Oss)	**Hetero**	**Hetero**	**Hetero**	SD<24w/PD	DOD
4	pT3aN0M1(Pul)	Major	Major	Major	SD<24w/PD	DOD
2	pT3aN0M1(Pul)	Major	Major	Major	CR/PR/SD>24w	AWD
4	pT3aN0M1(Pul)	Major	Major	Major	CR/PR/SD>24w	AWD
2	pT1aN0M1(Pul, Oss)	Major	Major	Major	CR/PR/SD>24w	AWD
3	pT2aN0M1(Oss, Lym)	**Hetero**	**Hetero**	**Hetero**	SD<24w/PD	DOD
4	pT3bN0M1(Pul, Hep)	**Hetero**	**Hetero**	**Hetero**	SD<24w/PD	DOD
4	pT4N1M1(Pul, Hep, Oss, Lym)	**Hetero**	**Hetero**	**Hetero**	SD<24w/PD	DOD
4	pT3aN0M1(Pul, Oss)	Major	Major	Major	SD<24w/PD	DOD
4	pT4N0M1(Pul, Hep, Oss)	Major	Major	Major	SD<24w/PD	AWD

Major: Major homozygote, Hetero: Heterozygote, Minor: Minor homozygote.
CR/PR/SD>24 m: complete, partial, or stable with >24 weeks response.
SD<24 m/PD: stable disease for <24 weeks or progressive disease.
AWD: alive with disease, DOD: dead of disease, NED: no evidence of disease.
Metastatic lesions*; Hep; Liver, Lym; lymph node, Oss; Bone, Pul; Lung.

Supplementary Table 2: Immunostaining for PD-L1 in RCC

	Immunostaining for PD-L1				*p* value
	0	1+	2+	3+	
Major (*n* = 37)	*n* = 11	*n* = 10	*n* = 11	*n* = 5	0.0014
Minor/Hetero (*n* = 16)	*n* = 1	*n* = 2	*n* = 7	*n* = 6	
